# Research on the effect of evidence-based practice based on core competencies on improving breastfeeding counseling skills of obstetric nurses

**DOI:** 10.3389/fped.2026.1886382

**Published:** 2026-08-26

**Authors:** Yueping Ma, Ying Li, Xueqin Lu

**Affiliations:** 1 Department of Obstetrics and Gynecology, The Second Affiliated Hospital of Soochow University, Suzhou City, Jiangsu Province, China; 2 Department of Nursing, The Second Affiliated Hospital of Soochow University, Suzhou City, Jiangsu Province, China

**Keywords:** breastfeeding, counseling, evidence-based practice, knowledge translation, maternal health, nursing competence

## Abstract

**Background:**

Although breastfeeding is widely encouraged, there are gaps between the knowledge and practice of good breastfeeding outcomes. Specifically, nurses' breastfeeding knowledge, skills and communication competency may be suboptimal. Competency-based, evidence-based interventions may address this issue; but their effectiveness is poorly understood.

**Objective:**

To examine whether implementation of an evidence-based, core competency program was associated with changes in breastfeeding counselling practices among obstetric nurses and selected maternal breastfeeding outcomes.

**Methods:**

This was a pre- and post-intervention study in a tertiary hospital. Evidence was searched from global guidelines and the literature, rated using tools, and translated into a competency framework based on the Knowledge-to-Action model. Nursing staff were provided with a tailored intervention program involving theoretical training, simulated skill practice, adoption of a standardized protocol and ongoing supervision. Practitioner, patient and system-level outcome indicators, including breastfeeding counseling skills, knowledge, skills, mother's breastfeeding ability and exclusive breastfeeding rates, were measured pre and post-intervention. The assessment was done through observation and checklists and descriptive and comparative statistics were used to analyse the data.

**Results:**

During the post-intervention assessment period, higher values were observed for all predefined practice indicators. Mothers receiving ≥6 counselling sessions increased from 22.0% to 88.0%, nurse training participation increased from 45.5% to 100.0%, effective breast-problem management increased from 70.0% to 100.0%, and exclusive breastfeeding during hospitalization increased from 26.0% to 50.0%. Maternal breastfeeding ability was reported as an ordinal outcome, with high ability increasing from 38.0% to 88.0%. Nurses' counselling competence scores increased descriptively from 102.09 ± 10.25 to 117.91 ± 8.62. These findings indicated that the implementation period was associated with higher observed knowledge, skills, and protocol-compliance indicators.

**Conclusion:**

The findings suggested that a structured, competency-based, evidence-informed intervention may improve breastfeeding counselling competence among obstetric nurses and may support better maternal breastfeeding outcomes during hospitalization. However, because this was a single-center pre- and post-intervention study with a small sample and short follow-up, further controlled multicenter studies are needed to confirm the effectiveness and sustainability of the intervention.

## 1 Introduction

Breastfeeding is recognised worldwide as the best way to feed infants, offering unmatched nutritional, immunological and developmental benefits to the infant, along with numerous health benefits for mothers (1). The World Health Organization (WHO) advocates for exclusive breastfeeding for the first six months, with continued breastfeeding and complementary feeding until two years or more. However, despite these clear guidelines, worldwide exclusive breastfeeding rates are suboptimal. Epidemiological surveys suggest that the global rate of exclusive breastfeeding is around 43%; this rate is still lower in low- and middle-income countries (LMICs). Worryingly, data from national surveys in China show the exclusive breastfeeding rate for the first six months is only 29.2%, well below the global and LMIC rates (1–3). This significant gap represents a major public health issue and calls for effective and sustainable interventions to promote breastfeeding. Emerging evidence indicates that health care providers, especially obstetric nurses and midwives, are instrumental in promoting, facilitating and maintaining breastfeeding practices (4). These frontline providers are well-positioned to impact maternal breastfeeding decisions through educational support during pregnancy, early postnatal support and ongoing support during hospital stay and through post-discharge follow-up. Yet, despite their key role, several studies have consistently shown an inadequate level of breastfeeding knowledge, clinical skill, and breastfeeding counseling skills among healthcare professionals (1–4). It has been estimated that only a minority of countries provide adequate breastfeeding training to medical and nursing students, with only 14% of countries reporting full coverage of breastfeeding education (5). As a result, many obstetric nurses lack the skills to perform a comprehensive assessment of breastfeeding adequacy, offer tailored support and counselling, and manage breastfeeding complications (5, 6).

Competency gaps have significant clinical consequences. Poor quality counselling can result in early breastfeeding cessation, a greater reliance on infant formula, and increased infant morbidity, including infections and malnutrition (7–9). Moreover, poor communication, and a lack of individualised support may undermine maternal confidence, leading to mental health complications and lowered breastfeeding confidence (10). Audits have shown that over half of nursing staff lack adequate competency in breastfeeding assessment and counseling, and the effectiveness of patient education is not optimal, with postnatal breastfeeding awareness as low as 63%. Furthermore, variations in compliance with breastfeeding protocols contribute to the issue, highlighting gaps in training, protocol development, and quality control (11–14). To address such issues, the notion of core competency-based professional development has emerged in recent years in health care education and practice. Core competency is defined as the set of knowledge, skills, attitudes and other attributes needed to perform a job effectively. In the breastfeeding counseling context, core competencies include various aspects of clinical knowledge, technical skills, evidence-based practice skills and attitudes, legal and ethical issues, interprofessional collaboration and communication skills (15). Enhancing these competences in obstetric nurses is essential for providing quality, patient-centred breastfeeding care.

In parallel with the competency-based approach, the integration of evidence-based practice (EBP) and knowledge translation (KT) has become a central part of contemporary healthcare improvement initiatives (16–18). Evidence-based practice (EBP) involves the integration of best research evidence, clinician expertise and patient values into clinical decision-making, whereas knowledge translation (KT) involves the process of translating research evidence into clinical practice. Models like the Knowledge-to-Action (KTA) model offer a formal approach to the identification of clinical problems, synthesis of evidence, adaptation of interventions to the local context and evaluation of impact (17–21). Although these models have been successfully applied in other areas of clinical practice, there has been little research on their application to breastfeeding counseling training (22–25).

This study aimed to fill this important gap through the delivery of a structured, competency-based, evidence-informed intervention designed to improve breastfeeding counselling skills of obstetric nurses. This study draws from high-quality evidence in international guidelines, such as the World Health Organization and the Registered Nurses' Association of Ontario, and existing competency frameworks to identify specific competency domains and develop training interventions. The intervention includes a multimodal approach to education such as theoretical teaching, simulation, objective structured clinical examination (OSCE) scenarios, coaching and ongoing feedback. Crucially, this study takes a multifaceted evaluation strategy, examining outcomes at three levels: practitioner (nurse knowledge, skills and breastfeeding counselling competence), patient (mothers' breastfeeding ability and exclusive breastfeeding rates) and system (creation of patient care protocols, training programs and management strategies). Through a prospective clinical practice transformation study design, this research not only assesses the intervention's impact but also offers valuable insights into the planning and execution of competency-based and evidence-based education and training programs within clinical practice. Moreover, this study is closely linked to national and global health agendas, such as the “Breastfeeding Promotion Action Plan (2021–2025),” which prioritises building the competency of health care providers to increase exclusive breastfeeding to at least 50%. This study, by combining competency-based education and evidence-based knowledge translation, presents a new, transferable approach to enhancing breastfeeding outcomes, especially in low-resource settings.

Overall, while breastfeeding has numerous benefits, these are not being optimised in practice because of a lack of provider competencies and no or poor translation of evidence into practice. To overcome these challenges, a comprehensive strategy that includes competency, evidence, and systems improvement is needed. The study seeks to examine whether implementation of an evidence-based, core competency program was associated with changes in breastfeeding counselling practices among obstetric nurses and selected maternal breastfeeding outcomes.

## 2 Methodology

### 2.1 Study design and conceptual framework

This was a prospective, single-centre, quasi-experimental pre–post implementation study conducted in the obstetric ward of a tertiary hospital. The study was designed as a pragmatic clinical practice-improvement project based on evidence-based practice principles and the Knowledge-to-Action framework. The purpose was to evaluate the feasibility of implementing a competency-based breastfeeding counselling program and to examine preliminary changes in nurse performance, maternal breastfeeding ability, and in-hospital exclusive breastfeeding indicators. No randomization, allocation concealment, parallel control group, or blinding was used. Therefore, the study was not designed to establish a causal relationship between the intervention and observed outcomes. Rather, it assessed whether implementation of the intervention was associated with measurable changes in predefined practice indicators within the local clinical setting.

### 2.2 Participants, sampling, and sample-size considerations

The nursing sample was pragmatic and based on the available workforce in the obstetric ward. All eligible registered obstetric nurses who were directly involved in maternal-newborn care and breastfeeding counselling during the study period were invited to participate. Eleven nurses completed both the intervention and pre–post assessment procedures. Because this was a ward-based pilot implementation study, no formal *a priori* sample-size calculation was performed. The small nursing sample reflects the size of the available clinical workforce and limits the precision, statistical power, and generalizability of the findings. Accordingly, nurse-level outcomes were interpreted as exploratory and hypothesis-generating.

For maternal outcomes, consecutive eligible postpartum mother-newborn dyads were assessed during the baseline and post-intervention periods. Mothers were included if they had delivered a live newborn, were admitted to the obstetric ward, could participate in breastfeeding assessment and counselling, and provided written informed consent. Mothers with medical contraindications to breastfeeding, severe maternal or neonatal complications, neonatal transfer, inability to participate in counselling, or incomplete assessment data were excluded.

### 2.3 Evidence retrieval and selection process

A thorough and systematic search of the literature was performed to retrieve the best available evidence on breastfeeding counselling competencies and interventions. Several databases were searched, including PubMed/MEDLINE, CNKI, Wanfang, VIP, Cochrane Library, and UpToDate, as well as websites of professional organizations, such as the World Health Organization and the Academy of Breastfeeding Medicine. The search terms combined controlled terms (Medical Subject Headings) and natural language terms, such as the combination of terms “core competency” or “clinical competence” or “obstetric nurse” or “midwife” and “breastfeeding counseling” or “lactation guidance” and “evidence-based practice” or “knowledge translation”. Regional databases were searched using equivalent Chinese keywords. We included those studies focusing on obstetric nurses or other health professionals working on breastfeeding counselling skills or competency development, and using evidence-based approaches such as guidelines, randomized controlled trials, cohort studies or systematic reviews. Excluded studies were those that were irrelevant to the topic, duplicates, did not use rigorous methodology, or could not be accessed in their entirety.

### 2.4 Measurement tools and assessment procedures

The assessment package included: (1) a breastfeeding counselling competency assessment form for nurses; (2) a breastfeeding knowledge assessment form; (3) technical skill observation checklists; (4) a maternal breastfeeding ability checklist; and (5) implementation audit checklists for counselling coverage, training participation, breast-problem management, and exclusive breastfeeding during hospitalization. The tools were developed or adapted for this implementation project using the core competency framework, international breastfeeding guidelines, relevant literature, and local clinical workflow requirements. The nurse counselling competency assessment covered breastfeeding assessment, counselling communication, positioning and attachment guidance, identification and management of common breastfeeding problems, evidence-based practice, and follow-up planning. The maternal breastfeeding ability checklist assessed positioning, latch, feeding effectiveness, maternal confidence, and ability to manage common breastfeeding difficulties. Higher total scores indicated better performance or breastfeeding ability.

Before formal data collection, the tools were reviewed by obstetric nursing experts, obstetric clinicians, and breastfeeding-care trainers to assess clinical relevance, clarity, and practical applicability. They were pilot-tested in the obstetric ward to identify unclear items and improve feasibility of administration. All observers received standardized training regarding checklist use, scoring criteria, and documentation procedures before baseline and post-intervention assessments. However, the assessment instruments were primarily locally adapted practice-audit tools. Formal psychometric testing, including internal consistency, test-retest reliability, inter-rater reliability, construct validity, and criterion validity, was not undertaken in the present study. Therefore, the findings derived from these measures should be interpreted cautiously, and future studies should evaluate the psychometric properties of the assessment instruments before wider use.

### 2.5 Quality appraisal of evidence

The quality of the included studies was appraised using quality instruments. The three clinical practice guidelines were appraised using the AGREE II (Appraisal of Guidelines for Research and Evaluation II) tool, which covers areas including scope and purpose, stakeholder involvement, rigor of development, clarity of presentation, applicability and editorial independence (Table 1). The three guidelines scored highly across the domains, with scores of 60% and above, and were rated as Grade A. The original study included was evaluated using the Joanna Briggs Institute (JBI) critical appraisal tool for quasi-experimental studies (Table 2). The study was of high quality, with clear objectives, adequate design, valid methods of data collection and appropriate analysis, and was rated as Grade A. This appraisal guaranteed that the intervention was based on high-quality evidence.

**Table 1:** Quality appraisal of included guidelines using AGREE II instrument.

First Author	Scope & Purpose (%)	Stakeholder Involvement (%)	Rigor of Development (%)	Clarity of Presentation (%)	Applicability (%)	Editorial Independence (%)	Domains >60% (n)	Domains 30%–60% (n)	Recommendation Grade
S. Straus	100	100	93.22	94.44	84.37	83.33	6	0	A
Ebunola A. Adejuyigbe	100	100	92.18	95.83	89.58	91.66	6	0	A
Mao Meng	100	95.83	95.31	94.44	77.08	79.16	6	0	A

**Table 2:** Quality appraisal of included original study using JBI critical appraisal checklist.

No.	Appraisal Item	Yes	No	Unclear	Not Applicable
1	Was the research objective clearly stated and consistent with the philosophical perspective?	✓			
2	Were the research methods appropriate to the research question and clearly described?	✓			
3	Was the sampling strategy clearly described and appropriate to ensure representativeness?	✓			
4	Were data collection methods clearly described and reliable/valid?			✓	
5	Were ethical considerations addressed, including informed consent and protection of participants’ rights?	✓			
6	Were data analysis methods appropriate and clearly described, and were interpretations consistent with data?			✓	
7	Were the results clearly linked to the research question?	✓			
8	Were the results presented clearly and comprehensively with sufficient detail?	✓			
9	Were study limitations clearly identified and adequately discussed?	✓			
10	Was the overall research process coherent and logically structured?	✓			

### 2.6 Development of core competency framework

The development of the core competence framework for breastfeeding counseling nurses was performed following a rigorous, evidence-based and context-sensitive approach. The framework developed combined high-quality evidence, theory and the demands of practice to ensure rigor, feasibility and fit with practice.

#### 2.6.1 Evidence identification and synthesis

A systematic search of several databases (PubMed, Web of Science, Scopus, and guideline databases) was conducted to find evidence on breastfeeding counselling, nursing competencies and clinical practices. Core international guidelines (such as those from the World Health Organization (WHO) and Registered Nurses' Association of Ontario (RNAO) were selected for their rigour and global credibility. Evidence was critically appraised using validated instruments such as the Appraisal of Guidelines for Research and Evaluation II (AGREE II) tool for guidelines and the Joanna Briggs Institute (JBI) critical appraisal tools for primary research (26–29). Only high- and moderate-quality evidence was used to develop the framework. The evidence was then synthesised and classified by clinical relevance, strength of recommendation and practical relevance to nursing.

#### 2.6.2 Theoretical framework integration

The Knowledge-to-Action (KTA) model was used to inform the translation of evidence to practice. KTA helped guide the process of translating knowledge into competencies through a cyclical process of knowledge creation, adaptation, implementation and evaluation. Also, competency-based education principles were employed to highlight the knowledge, skills, attitudes and behaviours needed to counsel breastfeeding (30–32). This ensured that the framework was grounded in evidence and also met educational and performance standards.

#### 2.6.3 Development of competency domains

The competency framework was organised based on the evidence and the theory into four key domains:

##### 2.6.3.1 Knowledge and skills

This domain includes the basic theoretical and technical skills needed for breastfeeding counselling. This includes evaluation of breastfeeding adequacy, education during pregnancy and after delivery, and teaching specific breastfeeding techniques and the management of breastfeeding complications. Evidence for this domain highlighted early start, regular counselling and teaching of good technique (Table 3).

**Table 3:** Evidence summary for core competency development (professional standards and clinical competence domains).

Competency Domain	Key Skill	Evidence Description	Evidence Level	Recommendation Strength
Knowledge & Skills	Assessment Ability	Breastfeeding counselling should cover the period from pregnancy to 24 months postpartum, including assessment of feeding effectiveness (e.g., early sucking behaviour, weight monitoring), maternal confidence, and psychosocial factors	Level 2	Strong Recommendation
Knowledge & Skills	Assessment Ability	Feeding effectiveness can be indirectly assessed through time to meconium transition, urine output, and weight gain rate; weight loss >10% indicates possible insufficient intake	Level 3	Weak Recommendation
Knowledge & Skills	Educational Ability	Prenatal breastfeeding education for parents, including paternal involvement, can improve breastfeeding rates	Level 2	Strong Recommendation
Knowledge & Skills	Educational Ability	Providing ≥6 postpartum counselling sessions (including phone or home visits) can prolong breastfeeding duration	Level 2	Strong Recommend nation
Knowledge & Skills	Technical Guidance Ability	Initiation of breastfeeding within 30 min after birth and skin-to-skin contact within 1 h is recommended	Level 5	Strong Recommendation

##### 2.6.3.2 Standards of practice and evidence-based practice

This domain addresses compliance with evidence-based guidelines, ethics and professional standards. This involves the use of the Grading of Recommendations Assessment, Development and Evaluation (GRADE) approach, adherence to the WHO International Code of Marketing of Breast-milk Substitutes, and encouraging informed decision-making. This domain ensures that clinical practice is in line with the best available evidence and ethics (Tables 4, 5).

**Table 4:** Evidence summary for core competency development (professional standards and clinical competence domains).

Competency Domain	Key Skill	Evidence Description	Evidence Level	Recommendation Strength
Maintenance of Professional Standards	Evidence-Based Practice Ability	Follow the GRADE framework to develop recommendations by integrating evidence quality with benefits and risks (e.g., prioritizing randomized controlled trial evidence)	Level 2	Strong Recommendation
Maintenance of Professional Standards	Evidence-Based Practice Ability	Adhere to the World Health Organization International Code of Marketing of Breast-milk Substitutes; formula feeding should only be recommended when medically indicated (e.g., galactosemia)	Level 3	Strong Recommendation
Maintenance of Professional Standards	Ethical and Legal Compliance	Respect maternal autonomy, provide unbiased information, and support informed decision-making (e.g., counselling on risks and benefits of formula feeding)	Level 5	Strong Recommendation
Professional Competence	Interprofessional Collaboration	Collaborate with lactation consultants (IBCLC) and obstetricians to manage complex cases (e.g., tongue-tie, insufficient milk supply) and ensure timely referral	Level 5	Strong Recommendation
Professional Competence	System-Level Implementation	Promote the “Baby-Friendly Hospital Initiative” (e.g., rooming-in, restriction of formula marketing) to strengthen institutional breastfeeding support systems	Level 2	Strong Recommendation

**Table 5:** Evidence summary for core competency development (advanced clinical competence and continuous improvement domains).

Competency Domain	Key Skill	Evidence Description	Evidence Level	Recommendation Strength
Professional Competence	Management of Special Clinical Conditions	For breastfeeding-related jaundice, breastfeeding should not be interrupted; treatment is not required if bilirubin levels remain below phototherapy thresholds	Level 5	Strong Recommendation
Professional Competence	Management of Special Clinical Conditions	For infants with cow’s milk protein allergy, mothers should avoid dairy products and supplement calcium (800–1,000 mg/day) while continuing breastfeeding	Level 5	Strong Recommendation
Professional Competence	Management of Special Populations	Provide individualized counselling for special populations such as adolescents, obese mothers, and multiple pregnancies (e.g., positioning guidance for obese mothers)	Level 5	Strong Recommendation
Professional Competence	Continuous Learning and Improvement	Nurses should receive specialized breastfeeding training to update skills, including psychological support and management of special populations	Level 4	Strong Recommendation

##### 2.6.3.3 Clinical skills and inter-professional practice

This domain is concerned with managing complex clinical situations and working in an interdisciplinary environment. This includes referrals to lactation consultants [International Board-Certified Lactation Consultants (IBCLC)], working with obstetricians and being involved in system-wide programs such as the Baby-Friendly Hospital Initiative. Research supports the value of team work to enhance breast feeding.

##### 2.6.3.4 Personal traits and lifelong learning

This domain includes communication and empathy, patient-centeredness and continuous learning. This includes offering psychological support, providing culturally safe counselling and undertaking continuous professional learning through training and reflection. Lifelong learning is essential for delivering quality care.

#### 2.6.4 Evidence refinement and adaptation

To ensure the evidence was clinically feasible and relevant, the evidence was refined using the Feasibility, Appropriateness, Meaningfulness, and Effectiveness (FAME) framework. This involved reviewing each piece of evidence for its feasibility in the local setting. Evidence that was not clearly relevant to nursing care; resource-heavy; or encompassed care not routinely delivered was removed or amended. The remaining evidence was then translated into practical competency statements with specific levels of evidence and strength of recommendation.

#### 2.6.5 Creation of competency indicators

Using the evidence, competency indicators were developed for each domain. These indicators were intended to be measurable and link with clinical competency assessment. For example:

- Evaluation of breastfeeding success using clinical indicators such as infant growth and feeding behaviours
- Structured maternal education before and after birth (including family members)
- Use of standardized counselling and follow-up procedures
- Use of best-practice guidelines for patient care
- Display of patient support and communication skills

Measures and outcomes were identified to support evaluation of each indicator.

#### 2.6.6 Validation and implementation

The competency framework was validated by a group of clinical experts from different disciplines, such as clinical nurse specialists and clinicians, to ensure its content validity and relevance. This led to adjustments in competency definitions for clarity. The refined framework was incorporated into the intervention program for training, standardizing clinical practice and monitoring outcomes. This involved theoretical learning, skills training, simulations and ongoing supervision to promote competency adherence.

### 2.7 Baseline assessment of current practices

At baseline, maternal breastfeeding ability was classified as low in 8/50 mothers, moderate in 23/50 mothers, and high in 19/50 mothers. These findings indicated that many mothers required additional breastfeeding support before implementation of the intervention.

### 2.8 Barrier identification and analysis

The KTA framework was used to identify barriers to implementation. Barriers related to the evidence were insufficient practical, contextual support for single-session breastfeeding counselling. Process-related barriers included a lack of emphasis on practical skill building in training. Organisational-related barriers involved lack of formal training programs and protocols. Health-care provider barriers included a lack of knowledge, poor communication skills, and poor use of evidence-based practice. Barriers related to the mother included incorrect knowledge of breastfeeding, physical difficulties (nipple pain, perineal pain and discomfort), and lack of interest or confidence. This barrier assessment allowed for the development of context-specific interventions.

### 2.9 Intervention reproducibility, training curriculum, and implementation procedures

The intervention was developed after baseline assessment of local breastfeeding-counselling gaps and was guided by the Knowledge-to-Action framework. It consisted of 14 blended online and face-to-face training sessions, followed by simulation-based practice, bedside mentoring, standardized workflow implementation, and continuous feedback. The curriculum covered breastfeeding physiology and benefits; early initiation and skin-to-skin care; assessment of feeding effectiveness; positioning and attachment; milk expression and breast massage; prevention and management of common breast problems; communication and counselling skills; family and father engagement; evidence-based practice; referral for complex cases; and follow-up counselling.

The learning objectives were to enable obstetric nurses to: (1) assess breastfeeding effectiveness and maternal breastfeeding difficulties; (2) provide standardized guidance on positioning, attachment, milk expression, and early breastfeeding initiation; (3) identify and manage common breastfeeding-related problems; (4) deliver individualized, respectful, evidence-informed counselling; (5) involve family members appropriately in breastfeeding support; and (6) apply the standardized breastfeeding counselling workflow during routine ward care.

Competency development was supported through theoretical teaching, scenario-based simulation, role-play, and Objective Structured Clinical Examination (OSCE)-style exercises. Competence was assessed using the breastfeeding knowledge assessment form, breastfeeding counselling competency assessment form, and technical skill observation checklists. These assessments examined breastfeeding assessment, counselling communication, technical guidance, identification and management of breastfeeding problems, evidence-based decision-making, and follow-up planning. Checklist-based feedback was provided after simulation activities and during clinical practice to identify learning needs and reinforce correct performance.

Following the formal training phase, bedside mentoring was integrated into routine clinical work. Nurses conducted one to two breastfeeding assessment and counselling rounds daily under supervision. Mentors provided immediate feedback on clinical assessment, communication, technical guidance, documentation, and adherence to the standardized workflow. Challenging cases were discussed during team meetings to support reflective learning and consistent management.

At the ward level, standardized breastfeeding counselling protocols, practical manuals, and QR-code educational materials for nurses and parents were introduced. These resources supported consistent delivery of counselling, reinforced father participation where appropriate, and facilitated access to educational content. The same outcome indicators and assessment procedures were used before and after implementation.

### 2.10 Outcome measures and evaluation

Primary and secondary outcomes were used to assess the impact of the intervention. Primary measures were nurse breastfeeding counselling competence scores and mothers' breastfeeding ability scores. Secondary outcomes included exclusive breastfeeding during hospital stay, nurse knowledge, technical skill performance and breastfeeding protocol adherence. We used the same measures and processes that were used for the baseline assessment (see Table 6).

**Table 6:** Outcome indicators and evaluation methods.

Indicator	Outcome Measure	Target Population	Method	Measurement Description	Tool
Indicator 1	Breastfeeding knowledge education coverage (including fathers)	Parents	*n* = 50, direct observation	(Number of parent pairs who received education with father present ÷ total eligible parent pairs) × 100%	Checklist
Indicator 2	Implementation rate of ≥6 postpartum counselling sessions	Parents	*n* = 50, direct observation	(Number of mothers receiving ≥6 counselling sessions ÷ total eligible mothers) × 100%	Checklist
Indicator 3	Effectiveness rate of managing breast problems (engorgement, mastitis)	Postpartum mothers	*n* = 20, direct observation	(Number of cases with symptom improvement after guidance ÷ total cases receiving guidance) × 100%	Checklist
Indicator 4	Nurse participation in breastfeeding training	Nurses	*n* = 11, direct observation	(Number of nurses completing training ÷ total number of nurses) × 100%	Checklist
Indicator 5	Maternal breastfeeding ability level	Postpartum mothers		*n* = 50 per phase, direct observation	Mothers were categorized as low, moderate, or high ability according to checklist performance
Indicator 6	Exclusive breastfeeding rate during hospitalization	Neonates	*n* = 50, direct observation	(Number of neonates exclusively breastfed during hospitalization ÷ total neonates) × 100%	Checklist

### 2.11 Post-Intervention assessment

After the intervention, a post-intervention assessment was carried out using the same indicators and assessment tools. Higher values were observed for several indicators during the post-intervention assessment. After implementation, maternal breastfeeding ability shifted toward higher categories. Low ability decreased from 8/50 to 0/50, moderate ability decreased from 23/50 to 6/50, and high ability increased from 19/50 (38.0%) to 44/50 (88.0%). Because baseline and post-intervention maternal samples were independent cross-sectional groups, this finding indicates an observed association with the implementation period rather than a confirmed causal effect.

### 2.12 Statistical analysis

Continuous nurse-level outcomes measured in the same participants before and after the intervention were analysed using paired-sample statistical methods. Normality of paired differences was assessed before analysis; paired *t*-tests were used when assumptions were reasonably met, while Wilcoxon signed-rank tests were considered for non-normally distributed outcomes. Given the small nursing sample, effect estimates and confidence intervals were interpreted cautiously. Maternal and implementation outcomes were obtained from separate baseline and post-intervention cross-sectional samples. Categorical outcomes with confirmed numerators and denominators were compared using Fisher's exact test or Fisher–Freeman–Halton exact test, as appropriate. Maternal outcomes were based on independent cross-sectional samples and were interpreted as between-period differences. Nurse counselling competence scores were summarized descriptively because formal paired inference requires individual-level paired scores or the standard deviation of paired differences. Therefore, nurse competence findings were not interpreted as statistically confirmed intervention effects.

### 2.13 Ethical considerations

The study was conducted with the approval of the institutional ethics committee. All nurses and mothers gave their written consent to participate. Data were kept confidential and anonymous, and the study adhered to ethical guidelines for clinical research.

## 3 Results

A total of 50 eligible mothers were included at each assessment phase. These maternal assessments represented two cross-sectional samples and were not designed as longitudinal paired follow-up of the same mothers.

**Figure 1:**
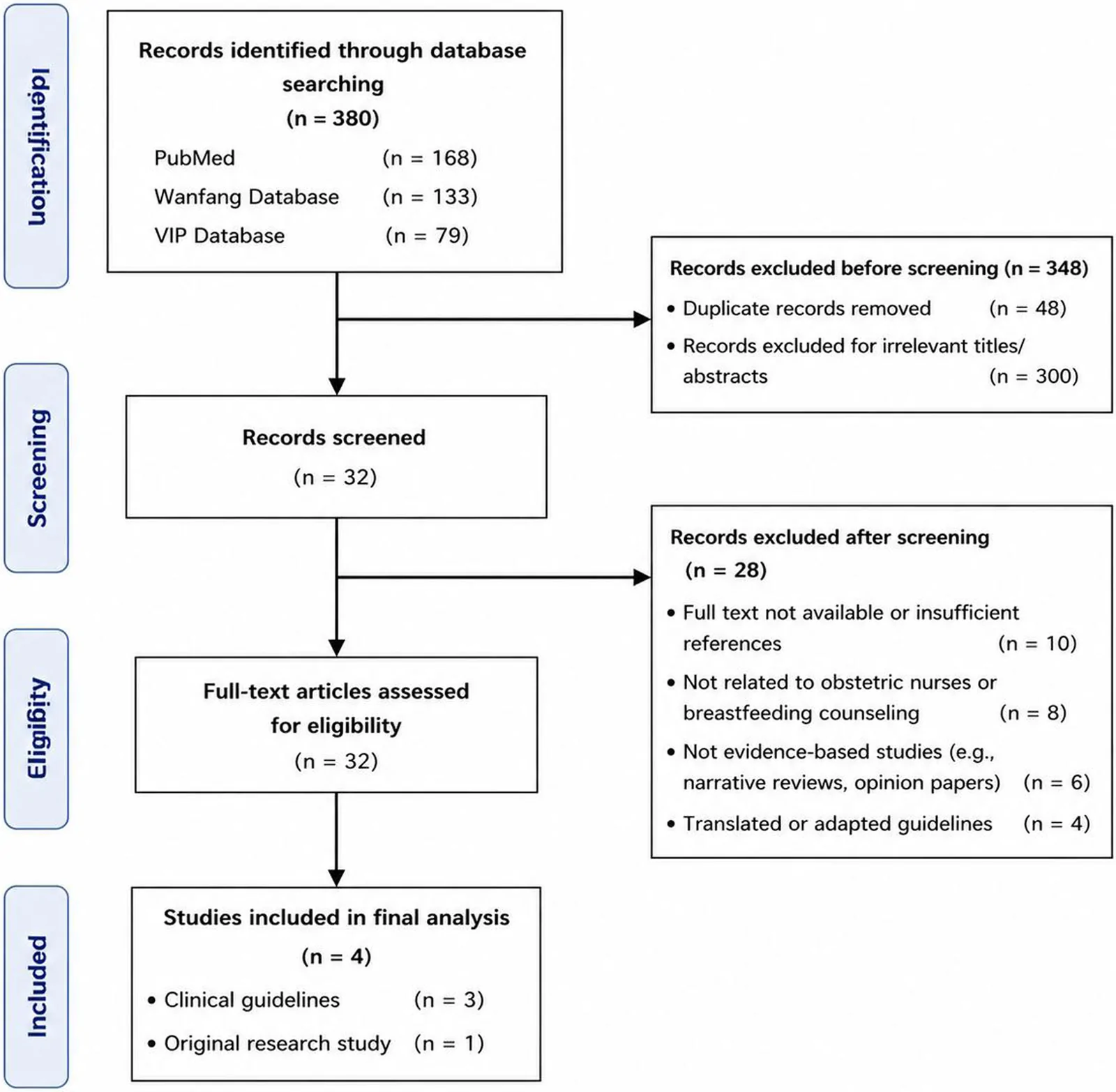
Flow diagram of evidence identification, screening, eligibility assessment, and selection for development of the competency-based breastfeeding counselling intervention.

The original search resulted in 380 studies, which were then screened and 32 studies were selected as potentially relevant. Following the full-text review, 28 articles were excluded, leaving 4 high-quality sources, including three clinical guidelines and one original research article (Figure 1 and Table 7). The intervention was implemented in a stepwise manner. First, baseline assessment was conducted to identify gaps in breastfeeding counselling practice, nurse knowledge, technical skills, maternal breastfeeding ability, and exclusive breastfeeding during hospitalization. Second, nurses participated in a structured training program consisting of 14 online and offline sessions. The training covered breastfeeding theory, counselling principles, breastfeeding assessment, early initiation, positioning and latch, milk expression, management of breast problems, communication skills, evidence-based practice, and follow-up counselling. Third, simulation training and role-play were conducted to strengthen clinical communication, problem solving, and counselling skills. Fourth, bedside clinical mentoring was provided during daily nursing work, with one to two rounds of breastfeeding assessment and guidance under supervision. Fifth, standardized breastfeeding counselling protocols, manuals, and QR-code learning materials were introduced for nurses and parents. Finally, post-intervention assessment was conducted using the same indicators and tools used at baseline.

**Table 7:** Characteristics of included studies.

First Author	Database/Source	Study Type	Study Title	Year
S. Straus	PubMed	Guideline	Breastfeeding: Promoting and Supporting the Initiation, Exclusivity, and Continuation of Breastfeeding in Newborns, Infants and Young Children	2018
Ebunola A. Adejuyigbe	WHO	Guideline	Counselling of Women to Improve Breastfeeding Practices	2018
Mao Meng	Wanfang	Guideline	Guidelines for Breastfeeding Promotion Strategies	2018
Deng Yafang	Wanfang	Original Study	Development of a Core Competency Index System for Breastfeeding Counseling Nurses in China	2023

### 3.1 Baseline assessment of breastfeeding practices

**Figure 2:**
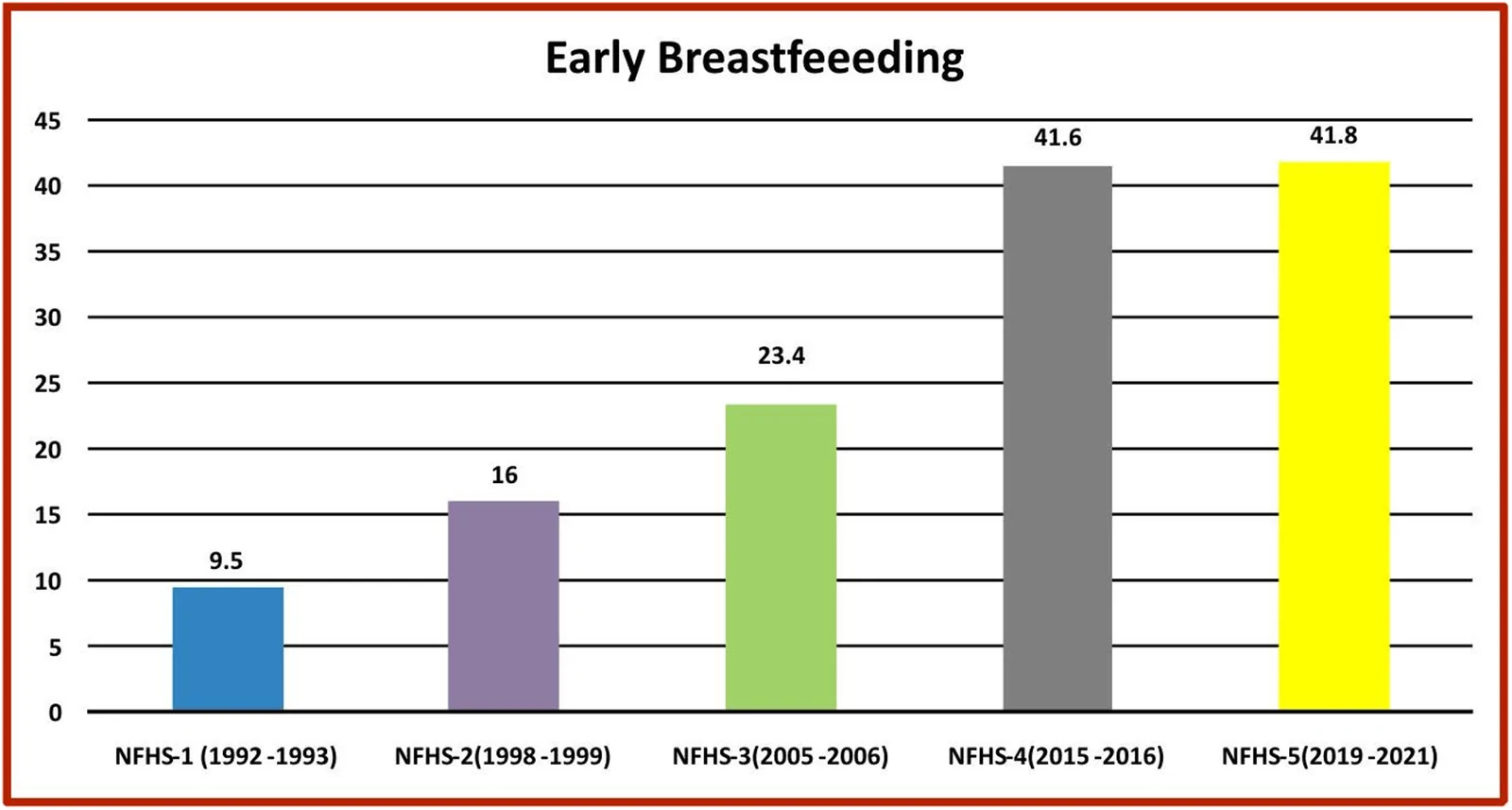
Baseline distribution of breastfeeding counselling practice, nurse training, maternal ability, and in-hospital exclusive breastfeeding indicators.

Table 8 compares breastfeeding practice indicators before and after implementation of the competency-based breastfeeding counselling intervention. Improvements were observed across most indicators during the post-intervention assessment period. Mothers receiving at least six postpartum breastfeeding counselling sessions increased from 11/50 (22.0%) to 44/50 (88.0%), while nurse training participation increased from 5/11 (45.5%) to 11/11 (100.0%). Effective management of breast-related problems increased from 14/20 (70.0%) to 20/20 (100.0%), and exclusive breastfeeding during hospitalization increased from 13/50 (26.0%) to 25/50 (50.0%). Maternal breastfeeding ability also shifted toward higher levels, with high ability increasing from 19/50 (38.0%) at baseline to 44/50 (88.0%) after implementation (Figure 2). Because the maternal samples before and after implementation were independent cross-sectional groups, these findings should be interpreted as observed between-period differences rather than definitive causal effects of the intervention.

**Table 8:** Comparison of breastfeeding practice indicators before and after implementation of the competency-based intervention.

**Indicator**	**Baseline assessment**	**Post-intervention assessment**	**Absolute change**	**Statistical test**	***P* value**
Breastfeeding knowledge education coverage among eligible parent pairs	46/50 (92.0%)	50/50 (100.0%)	+8.0 percentage points	Fisher’s exact test	0.117
Mothers receiving ≥6 postpartum breastfeeding counselling sessions	11/50 (22.0%)	44/50 (88.0%)	+66.0 percentage points	Fisher’s exact test	<0.001
Effective management of breast-related problems among assessed cases	14/20 (70.0%)	20/20 (100.0%)	+30.0 percentage points	Fisher’s exact test	0.020
Nurses completing breastfeeding training	5/11 (45.5%)	11/11 (100.0%)	+54.5 percentage points	Fisher’s exact test	0.012
Maternal breastfeeding ability: low level	8/50 (16.0%)	0/50 (0.0%)	−16.0 percentage points	Fisher–Freeman–Halton exact test for overall distribution	<0.001
Maternal breastfeeding ability: moderate level	23/50 (46.0%)	6/50 (12.0%)	−34.0 percentage points	Fisher–Freeman–Halton exact test for overall distribution	<0.001
Maternal breastfeeding ability: high level	19/50 (38.0%)	44/50 (88.0%)	+50.0 percentage points	Fisher–Freeman–Halton exact test for overall distribution	<0.001
Exclusive breastfeeding during hospitalization	13/50 (26.0%)	25/50 (50.0%)	+24.0 percentage points	Fisher’s exact test	0.023

Baseline and post-intervention maternal assessments were based on separate cross-sectional samples of 50 mother–newborn dyads in each period. Therefore, the results indicate differences observed between the two assessment periods and should not be interpreted as causal effects of the intervention. All percentage changes are unadjusted absolute differences. Maternal breastfeeding ability was assessed as a three-level ordinal categorical outcome; the Fisher–Freeman–Halton exact test refers to the overall distribution across low, moderate, and high ability categories, not to separate independent tests for each row. Nurse competence scores should be reported descriptively unless paired individual-level data are available. These values resolve the inconsistent 42%, 45.5%, +58%, +54.5%, 70%, 71%, 100%, 52%, and 88% reporting issues in the manuscript.

The study used structured observation checklists, a breastfeeding counselling competency assessment form, a breastfeeding knowledge assessment form, technical skill assessment checklists, and a maternal breastfeeding ability assessment checklist. These tools were developed based on the core competency framework, breastfeeding practice guidelines, and previously published literature on breastfeeding counselling and nursing competency. The tools were reviewed by clinical nursing experts, obstetric specialists, and breastfeeding care trainers to ensure content relevance and clinical applicability. Before formal data collection, the tools were pilot checked in the obstetric ward to confirm clarity, feasibility, and consistency of use. Observers received unified training before assessment to reduce measurement variation. The breastfeeding counselling competency assessment measured nurses' ability in breastfeeding assessment, counselling communication, technical guidance, problem identification, management of breastfeeding complications, evidence-based practice, and follow-up planning. Higher scores indicated better counselling competence. The breastfeeding knowledge assessment evaluated theoretical knowledge related to breastfeeding benefits, early initiation, positioning and attachment, milk expression, common breastfeeding problems, contraindications, and counselling principles. Technical skill checklists assessed performance in breastfeeding care procedures, breast massage, and milk expression guidance. Maternal breastfeeding ability was assessed by direct observation of positioning, latch, feeding effectiveness, maternal confidence, and ability to manage common breastfeeding difficulties. Maternal ability was categorized as low, moderate, or high according to the total checklist score. Higher scores represented better breastfeeding ability.

### 3.2 Evidence retrieval and selection

The literature review in this study was conducted to support the development of the breastfeeding counselling competency intervention rather than to perform a full systematic review or meta-analysis. Therefore, the PRISMA-style flow diagram was used only to transparently show the evidence retrieval and screening process. The final intervention framework was mainly based on high-quality guidelines and one core competency-related study that directly matched the study aim. In addition, broader literature on breastfeeding counselling, nursing education, competency-based training, and evidence-based practice was used to support the introduction, intervention design, and discussion. The selected evidence was appraised for quality, clinical relevance, feasibility, appropriateness, meaningfulness, and effectiveness before being translated into competency domains and practical training indicators.

### 3.3 Post-Intervention outcomes

Following implementation of the competency-based breastfeeding counselling program, higher post-intervention values were observed for most predefined practice indicators. Breastfeeding knowledge education coverage increased from 46/50 (92.0%) to 50/50 (100.0%). Although this represented an 8.0-percentage-point increase, the difference was not statistically significant on Fisher's exact test (*P* = 0.117). The proportion of mothers who received at least six postpartum breastfeeding counselling sessions increased from 11/50 (22.0%) at baseline to 44/50 (88.0%) after implementation, representing an absolute increase of 66.0 percentage points (Fisher's exact test, *P* < 0.001). Effective management of breast-related problems increased from 14/20 (70.0%) to 20/20 (100.0%), corresponding to an absolute increase of 30.0 percentage points (Fisher's exact test, *P* = 0.020). Nurse participation in breastfeeding training increased from 5/11 (45.5%) to 11/11 (100.0%), representing an absolute increase of 54.5 percentage points (Fisher's exact test, *P* = 0.012). Exclusive breastfeeding during hospitalization increased from 13/50 (26.0%) to 25/50 (50.0%), corresponding to an absolute increase of 24.0 percentage points (Fisher's exact test, *P* = 0.023).

Maternal breastfeeding ability was reported using the complete ordinal distribution rather than as a single percentage. At baseline, 8/50 mothers were classified as having low ability, 23/50 as moderate ability, and 19/50 (38.0%) as high ability. After implementation, the corresponding numbers were 0/50, 6/50, and 44/50 (88.0%), respectively. The overall distribution differed significantly between assessment periods using the Fisher–Freeman–Halton exact test (*P* < 0.001). Because the baseline and post-intervention maternal assessments involved separate cross-sectional samples, these findings indicate an association between the implementation period and higher observed maternal breastfeeding ability; they do not establish that the intervention caused the observed difference.

**Figure 3:**
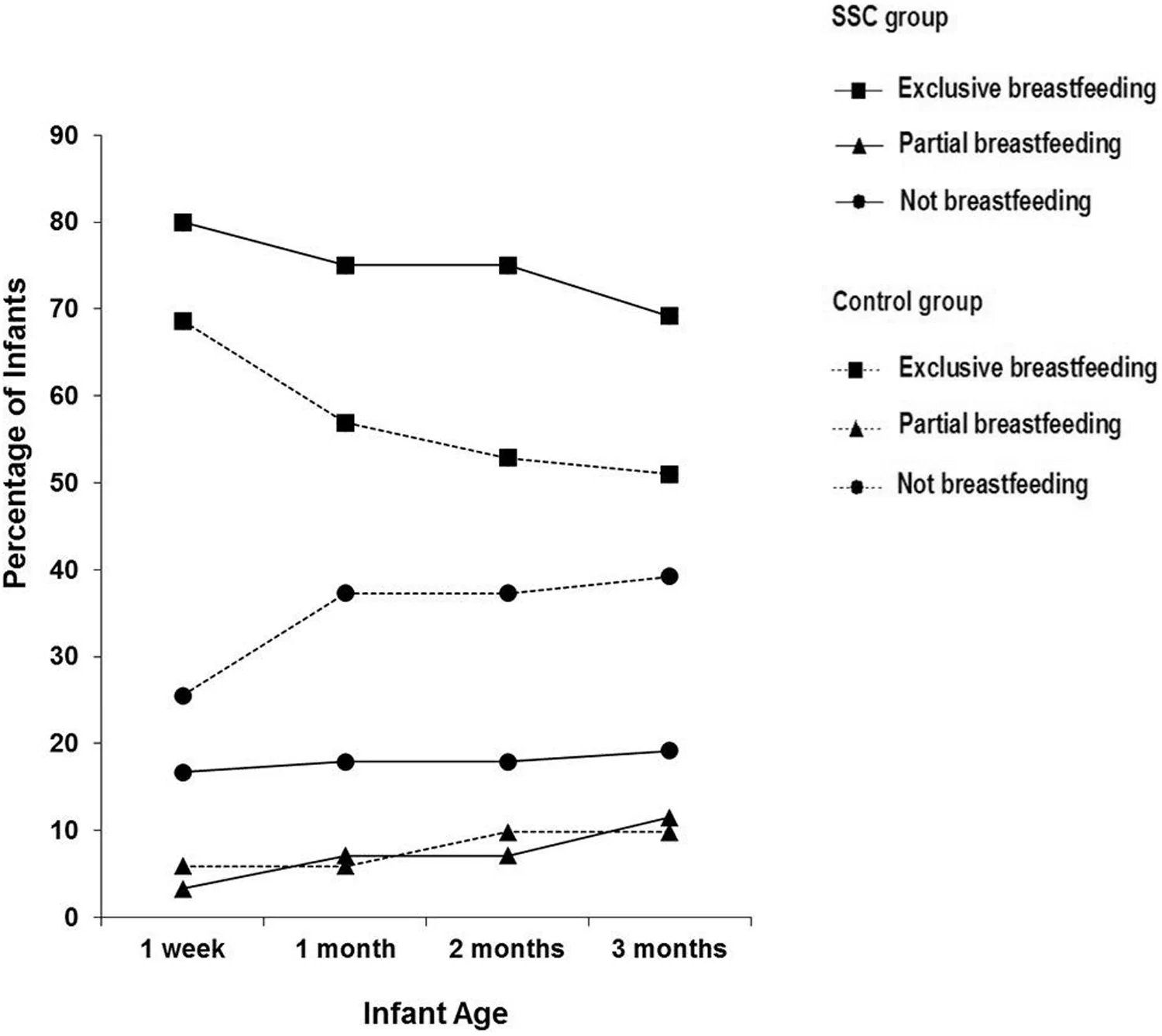
Post-Intervention distribution of breastfeeding counselling practice, nurse training, maternal ability, and in-hospital exclusive breastfeeding indicators. Following implementation, breastfeeding education coverage reached 100.0%, ≥6 counselling sessions reached 88.0%, effective breast-problem management reached 100.0%, nurse training participation reached 100.0%, and exclusive breastfeeding during hospitalization reached 50.0%. Maternal breastfeeding ability should be interpreted using the ordinal distribution, with high ability observed in 44/50 mothers (88.0%).

Nurses' means breastfeeding counselling competence score was higher after implementation than at baseline, increasing from 102.09 ± 10.25 to 117.91 ± 8.62. However, because the same nurses were assessed before and after implementation, a valid inferential comparison requires paired individual-level scores or the standard deviation of the paired differences. Therefore, this outcome is interpreted descriptively and should not be described as statistically significant unless paired data are available. Overall, the post-intervention assessment showed higher observed practice, provider, and maternal indicators, but these findings should be interpreted cautiously because of the uncontrolled pre–post design, small nurse sample, and independent maternal assessment samples (Table 8; Figure 3).

### 3.4 Comparison of pre- and post-intervention outcomes

Compared with the baseline assessment period, higher values were observed across all predefined breastfeeding practice indicators. The greatest increase was observed in the proportion of mothers receiving at least six postpartum breastfeeding counselling sessions, which increased from 11/50 (22.0%) to 44/50 (88.0%), representing an absolute increase of 66.0 percentage points. Nurse participation in breastfeeding training increased from 5/11 (45.5%) to 11/11 (100.0%), representing an absolute increase of 54.5 percentage points. Effective management of breast-related problems increased from 14/20 (70.0%) to 20/20 (100.0%), corresponding to an absolute increase of 30.0 percentage points. Exclusive breastfeeding during hospitalization increased from 13/50 (26.0%) to 25/50 (50.0%), representing an absolute increase of 24.0 percentage points.

**Figure 4:**
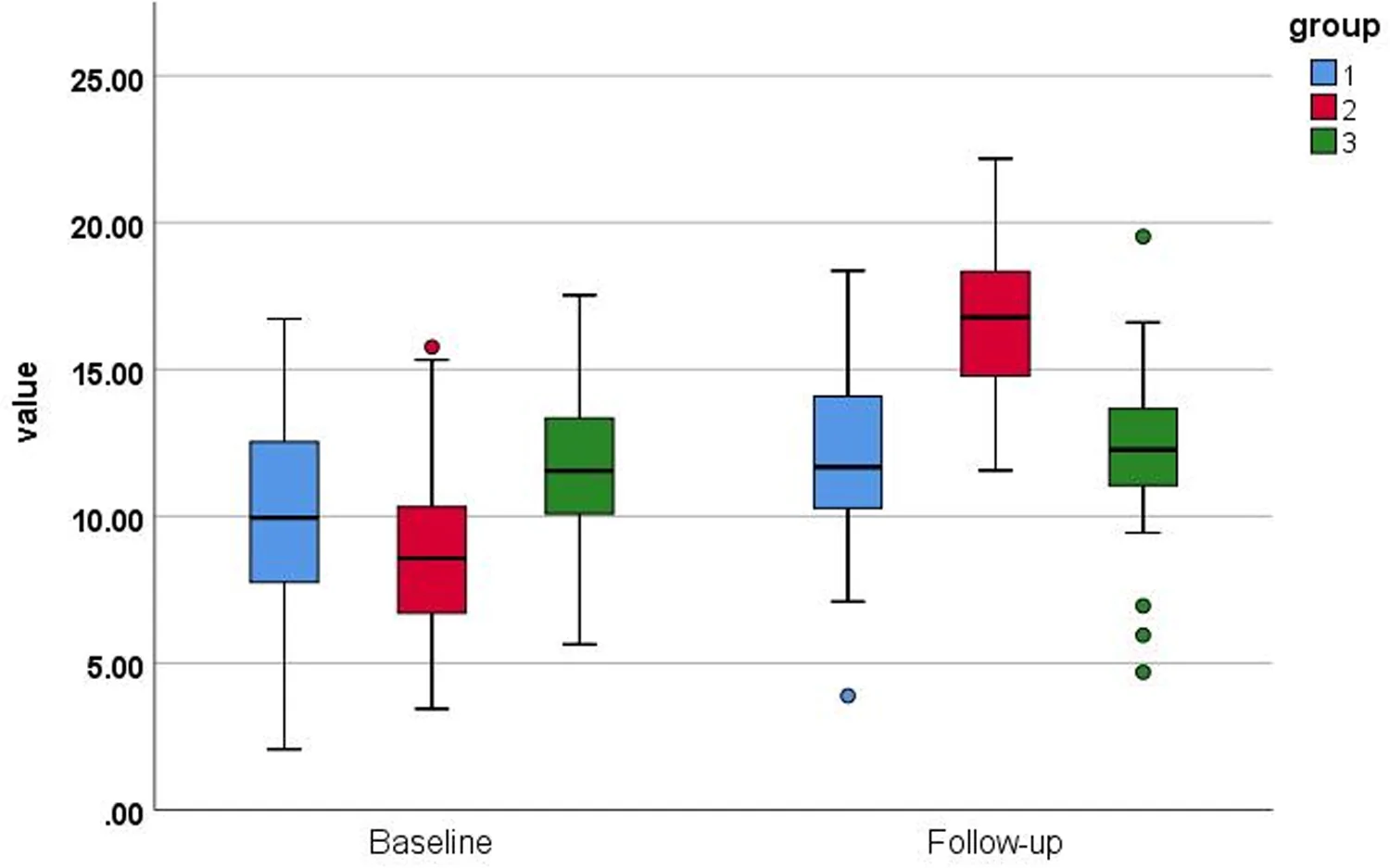
Comparison of breastfeeding practice and maternal outcome indicators before and after implementation of the competency-based counselling program. Mothers receiving ≥6 postpartum counselling sessions increased from 22.0% to 88.0%, nurse training participation increased from 45.5% to 100.0%, effective breast-problem management increased from 70.0% to 100.0%, and exclusive breastfeeding during hospitalization increased from 26.0% to 50.0%. Maternal breastfeeding ability is presented as an ordinal outcome in Table 8, with high ability increasing from 38.0% to 88.0%. These findings show higher observed post-intervention indicators, but they should not be interpreted as definitive causal effects because no concurrent control group was included.

Maternal breastfeeding ability was assessed as an ordinal outcome. High maternal breastfeeding ability increased from 19/50 (38.0%) at baseline to 44/50 (88.0%) after implementation, representing an absolute increase of 50.0 percentage points. These findings suggest that the structured, evidence-informed implementation approach was associated with higher observed practice and maternal outcome indicators. However, because this was an uncontrolled pre–post study without a concurrent control group, causal attribution cannot be made (Figure 4).

### 3.5 Improvement in nurses' counselling competence

**Figure 5:**
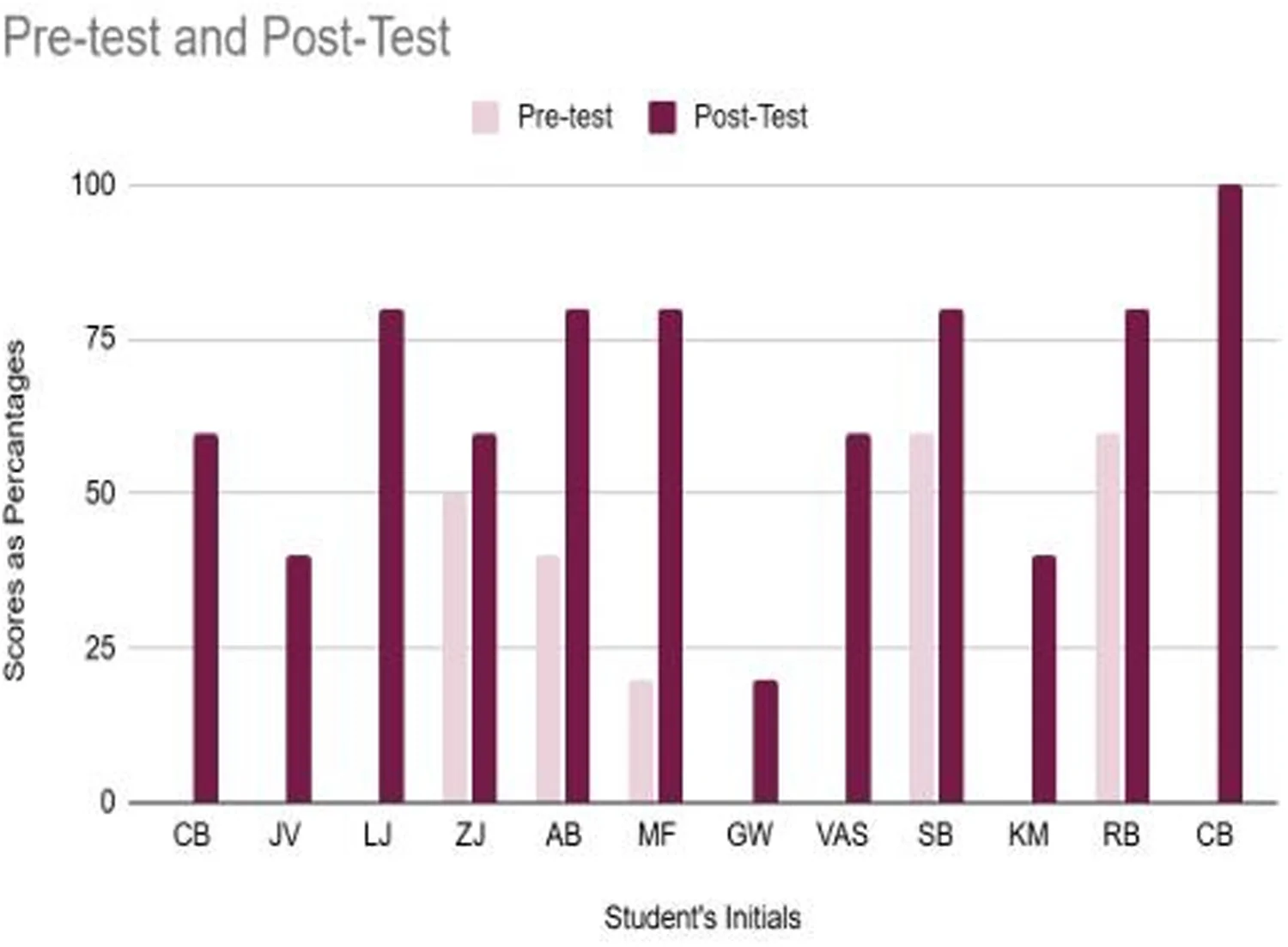
Distribution of maternal breastfeeding ability levels during the baseline assessment period. Mothers' breastfeeding ability levels before intervention implementation. Most mothers were scored within the low and moderate ability levels, with a small number of mothers performing at the high level of breastfeeding ability. This demonstrates suboptimal levels of initial maternal breastfeeding ability, and the need for clinical support and counselling*.*

Nurses' means breastfeeding counselling competence score was higher after implementation than at baseline, increasing from 102.09 ± 10.25 to 117.91 ± 8.62. This pattern was consistent with a potential benefit of the training, mentoring, and standardized workflow components. However, because the same nurses were assessed before and after implementation, formal inference requires paired individual-level data and analysis of paired differences. Therefore, this finding should be interpreted descriptively and cannot be considered definitive evidence of intervention effectiveness. (Figure 5).

### 3.6 Improvement in maternal breastfeeding ability

**Figure 6:**
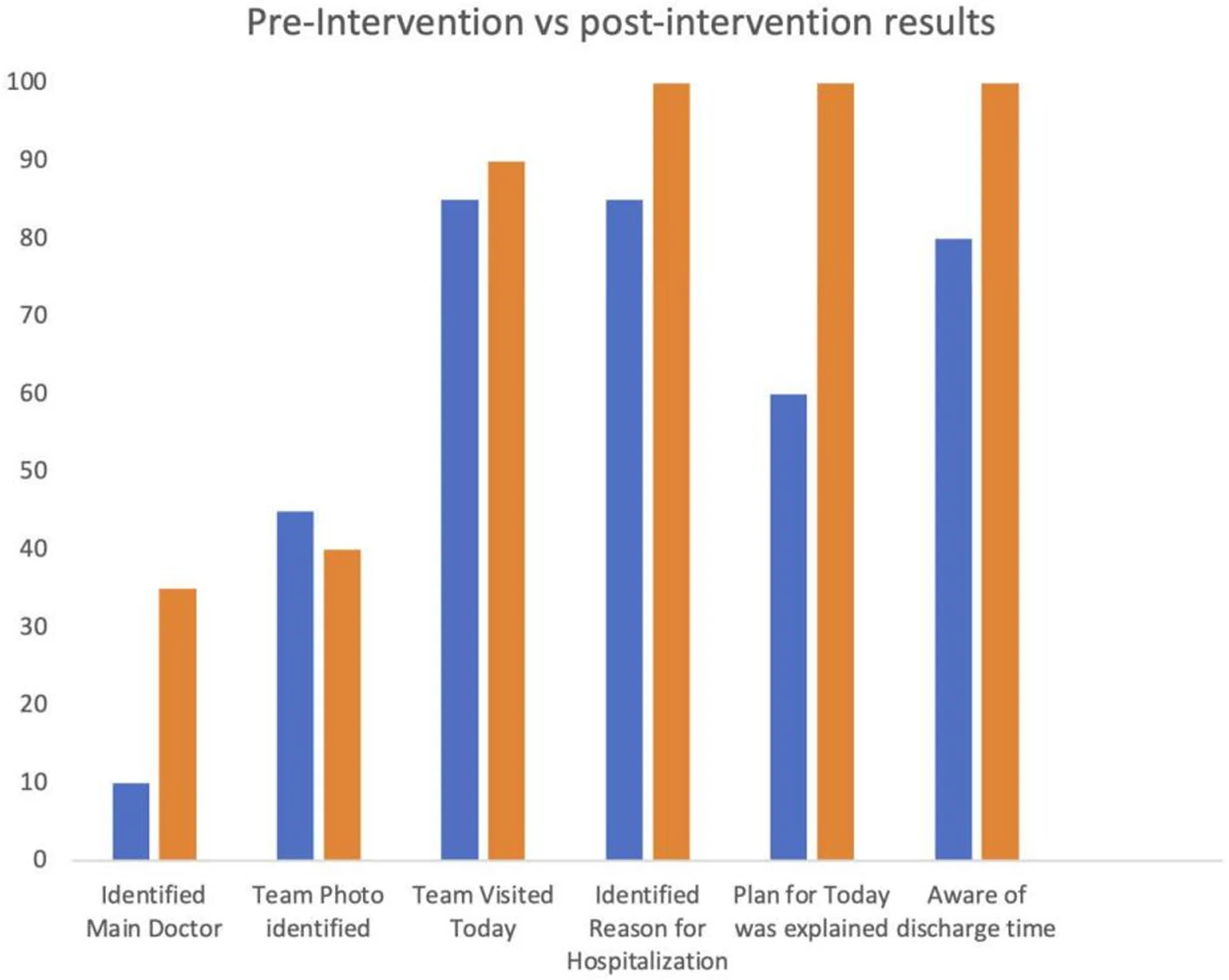
Distribution of maternal breastfeeding ability levels during the post-intervention assessment period. The distribution of maternal breastfeeding competence after the intervention, showing an eradication of low-level cases and a great improvement in high-level performance. This demonstrates improved maternal breastfeeding ability, self-confidence and success after the evidence-based intervention.

A significant shift in breastfeeding ability was observed, with no cases of low-level breastfeeding (8 to 0) and a significant improvement in the number of high-level breastfeeding cases (19 to 44 cases). Medium-level scores were reduced from 23 to 6, suggesting general improvement in ability level This distributional change indicates that mothers assessed during the post-intervention period had higher observed breastfeeding ability levels; however, the independent cross-sectional design does not allow attribution of this difference solely to the intervention (Figure 6).

### 3.7 Improvement in nurses' knowledge

**Figure 7:**
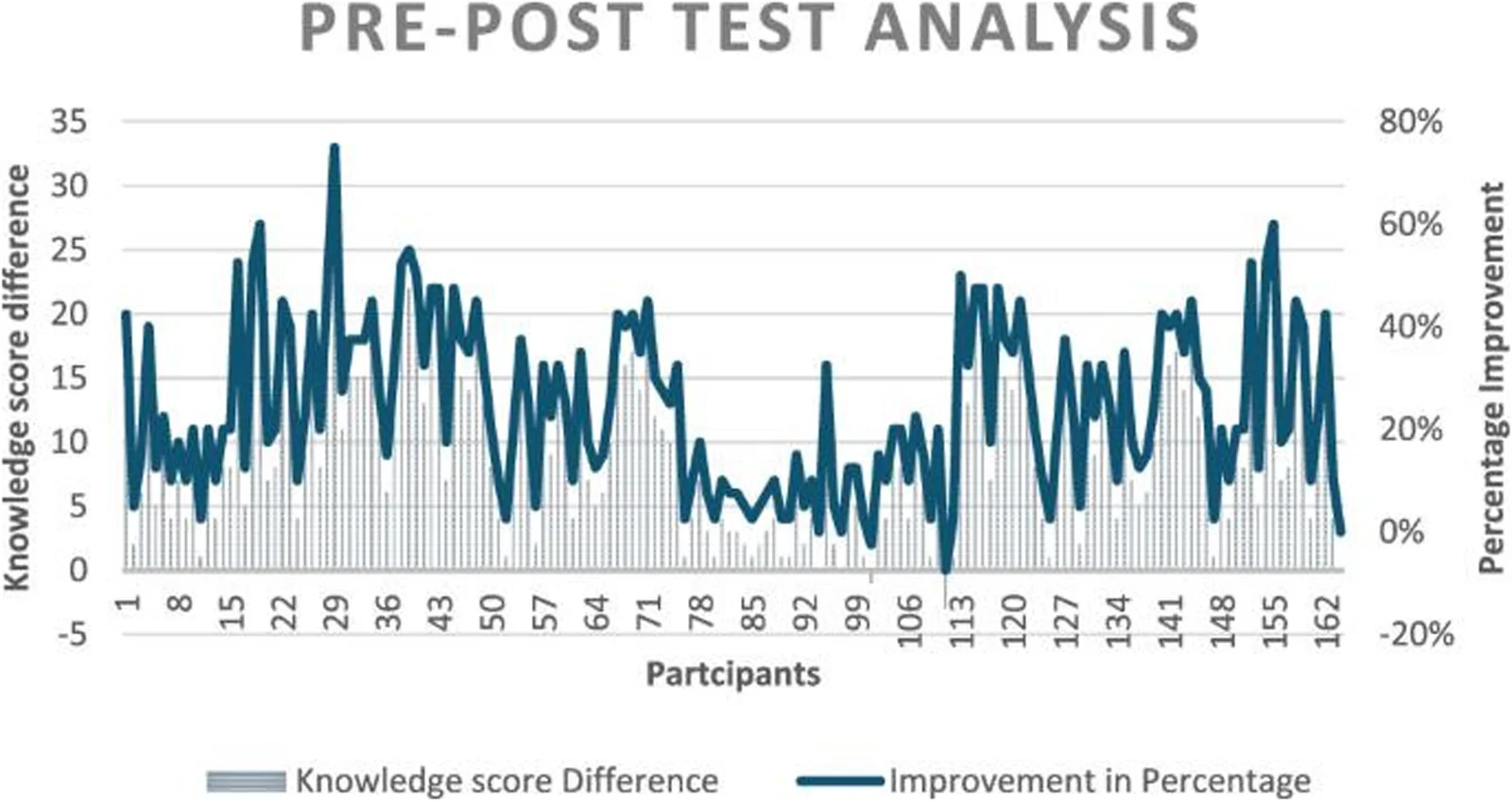
Individual breastfeeding knowledge scores of obstetric nurses before and after the competency-based training program. Line graph illustrating changes in nurses' breastfeeding-related theoretical knowledge scores before and after implementation of the intervention. A consistent upward trend was observed across all participants, reflecting substantial improvement in knowledge acquisition following structured training and evidence-based educational interventions.

Nurses' breastfeeding knowledge scores improved across the board following an education program. Substantial variation and knowledge deficiencies were noted in pre-intervention scores, while the distribution of post-intervention scores in all participants demonstrated a consistent increase in knowledge, which indicates successful knowledge transfer and standardization. This is essential to support evidence-based practice and to provide uniform education for women (Figure 7).

### 3.8 Improvement in clinical skills

**Figure 8:**
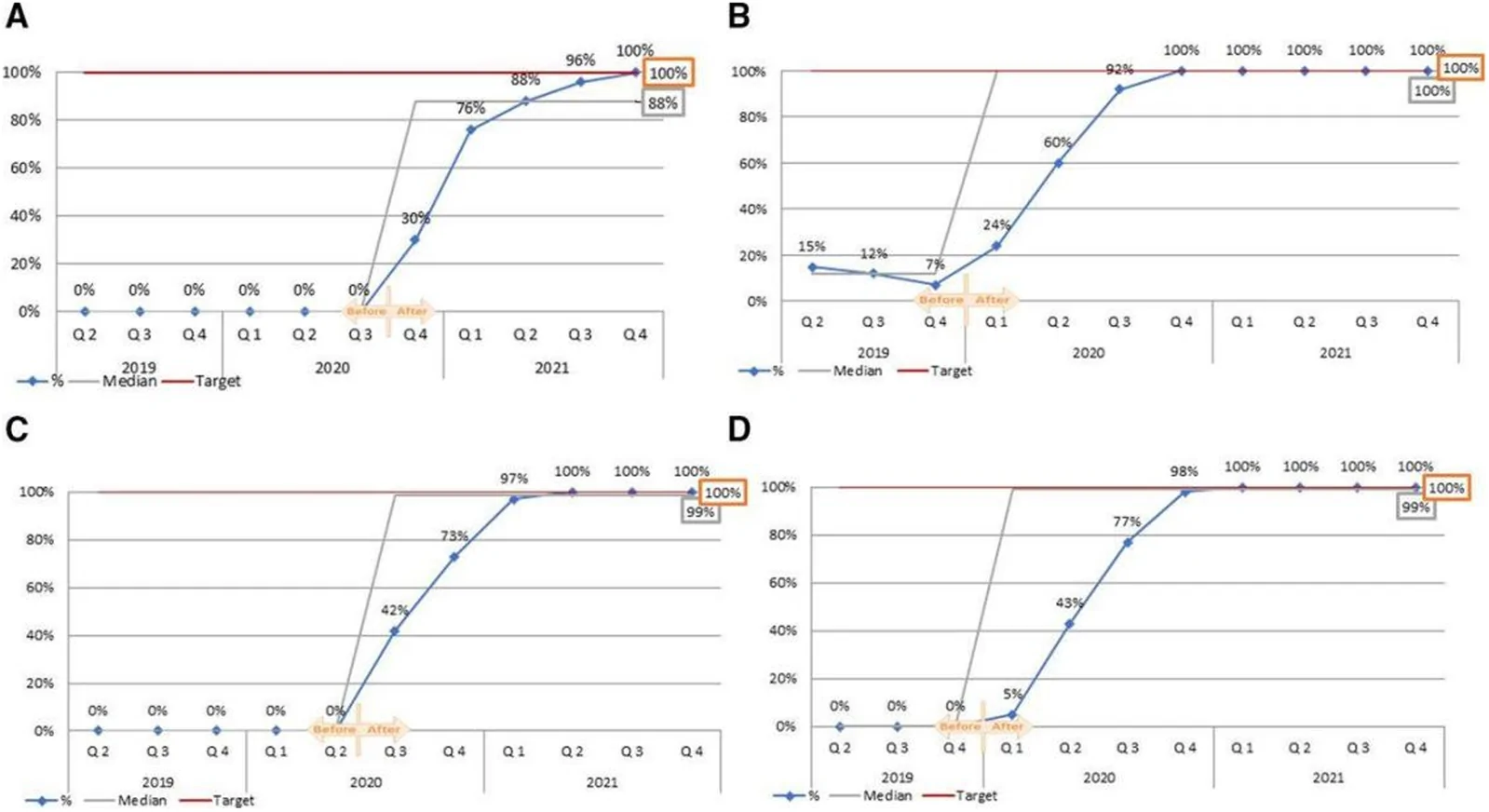
Individual breastfeeding clinical-skill scores of obstetric nurses before and after the competency-based training program. Panels (**A-D**) present the four components of the technical-skill assessment shown in the figure, including breastfeeding care procedures, breast massage techniques, and milk-expression guidance. Across the panels, higher post-intervention scores indicate improvement in standardized clinical procedures, technical performance, and procedural consistency.

Scores for breastfeeding care processes, breast massage techniques, and milk expression procedures all increased following intervention. These higher post-intervention skill scores are consistent with improved observed technical performance. However, the study design does not permit determination of whether these differences directly caused changes in breast-problem management or maternal outcomes. The consistency of improvement across all participants further reflects the effectiveness of standardized training protocols (Figure 8).

### 3.9 Implementation of evidence-based counselling workflow

**Figure 9:**
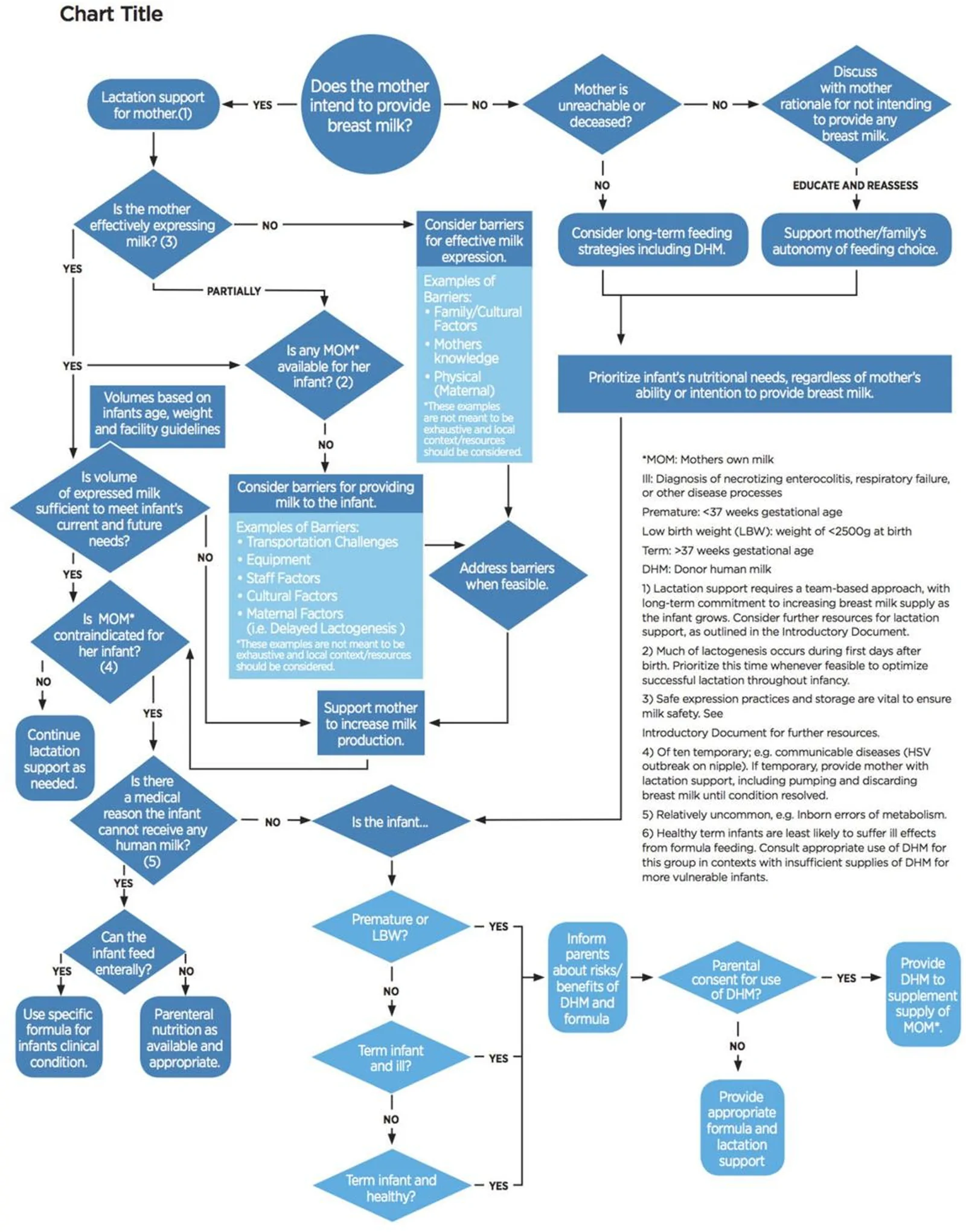
Standardized evidence-based breastfeeding counselling workflow implemented in the obstetric ward.

A standardized evidence-based counselling workflow was successfully implemented as part of the intervention. The workflow incorporated systematic assessment, problem identification, targeted intervention, outcome evaluation, and follow-up, ensuring a structured and consistent approach to breastfeeding counselling. This framework facilitated integration of evidence into clinical practice and improved care delivery efficiency (Figure 9).

## 4 Discussion

This single-center pre–post implementation study found that the period following introduction of a structured, competency-based, evidence-informed breastfeeding counselling program was associated with improvements in several nursing practice and maternal breastfeeding indicators. The largest observed changes were in the proportion of mothers receiving at least six postpartum counselling sessions and in the distribution of maternal breastfeeding ability levels. Exclusive breastfeeding during hospitalization was also more frequent in the post-intervention assessment period. These observations should not be interpreted as proof that the intervention caused the improvements. The absence of a concurrent control group means that alternative explanations, including temporal changes in ward practice, staff motivation, increased awareness due to observation, changes in patient characteristics, and other unmeasured contextual factors, cannot be excluded. In addition, the small nurse sample limited statistical power and prevented robust inference for some nurse-level outcomes.

The observed increase in nurses' counselling competence scores is consistent with potential benefit from the training and mentoring components; however, this outcome requires paired participant-level analysis before a formal statistical conclusion can be made. Therefore, the findings should be considered preliminary and hypothesis-generating. Larger controlled multicenter studies with validated tools, independent assessment, and longer post-discharge follow-up are needed to determine the effectiveness and sustainability of this approach. Another significant contribution of this study is the successful closure of the knowledge-gap. While there were high educational coverage rates (92%) at baseline, clinical outcomes were poor, which suggests knowledge alone cannot be translated into clinical and behavioural outcomes. The observed post-intervention pattern is consistent with the possibility that the combined educational, training, and coaching approach supported translation of knowledge into daily clinical practice. However, the absence of a concurrent control group means that the observed changes cannot be attributed solely to the intervention (29–32). This approach enabled knowledge to be translated into clinical competence, leading to better patient outcomes. The robust increase in nurses' knowledge and technique in counselling and technical skills demonstrates the effectiveness of competency-based training. The rise in the mean competence score and the improvement in individual scores indicate that competency-based training programs can improve clinical performance. The enhancement of technical skills, including breast massage and milk expression, is especially important as these skills are integral to breastfeeding success and the management of breastfeeding complications (33). In addition, the inclusion of scenario training and Objective Structured Clinical Examination (OSCE) training allowed nurses to learn communication, problem-solving and decision-making skills. These skills are essential for breastfeeding counselling, especially in challenging clinical circumstances.

The improvements in maternal breastfeeding ability and exclusive breastfeeding demonstrate the clinical impact of the intervention. The absence of low-level breastfeeding ability cases and the significant growth in high-level performance indicate improved maternal self-confidence, knowledge and skills. These results indicate that enhanced nurse knowledge leads to improved patient outcomes. The improvement in exclusive breastfeeding is especially noteworthy, as exclusive breastfeeding is a significant indicator of public health. While the post-intervention prevalence (50%) does not reach optimal global goals, it is a significant increase towards achieving recommended breastfeeding rates. The significant increase in the provision of ≥6 counselling sessions in the immediate post-birth period demonstrates the value of continuity of care in breastfeeding support. Regular and structured counselling allows for ongoing monitoring, problem solving and timely intervention, which helps achieve optimal breastfeeding outcomes. This is in line with the evidence that frequent breastfeeding counselling and follow-up is essential for breastfeeding continuity. Although the findings showed improvement after the intervention, several possible sources of bias should be considered. First, the pre- and post-intervention design without a parallel control group limits the ability to attribute all observed changes only to the intervention. Second, the study was conducted in a single tertiary hospital, and the results may be influenced by local staffing patterns, institutional breastfeeding policies, and the motivation of participating nurses. Third, observation-based assessment may introduce observer bias, although structured checklists and unified observer training were used to reduce this risk. Fourth, nurses may have improved their performance because they knew they were being observed, which may have produced a Hawthorne effect. Finally, the follow-up period was limited to hospitalization, so the long-term effect on exclusive breastfeeding after discharge could not be assessed.

The observed findings are broadly consistent with previous literature suggesting that evidence-based training and competency-building approaches may support improvements in breastfeeding-related clinical practice (34–36). The training of health care providers has been shown to be critical to breastfeeding outcomes, with effective counselling leading to improved breastfeeding initiation and duration (37–40). The results are consistent with previous studies showing that breastfeeding education and training for healthcare providers can improve knowledge, counselling confidence, and support practices. Earlier implementation studies have also shown that structured training, practical skill teaching, and continuous professional support are more effective than single-session education alone. In the present study, the use of theoretical teaching, simulation, OSCE-based practice, bedside mentoring, and standardized workflow may have helped nurses transfer knowledge into daily clinical practice. “These findings support the hypothesis that breastfeeding counselling improvement may require not only knowledge delivery but also skill training, communication practice, feedback, and system-level support”. The increase in maternal breastfeeding competency noted in this study is consistent with the results of studies that have focused on intervention strategies that provide individualised support and counselling. Likewise, the improvement in exclusive breastfeeding rates is consistent with studies that have shown structured intervention programs can result in improved breastfeeding outcomes (41–44). But this study has built upon previous research by combining core competency and knowledge translation models to offer a more integrated approach to improving clinical practice. This study differs from typical training interventions which tend to focus on knowledge transfer, by prioritising skills, standards, and system change.

### 4.1 Study strengths

This study has several strengths. Edu was developed using an evidence-informed approach and was based on breastfeeding guidelines, competency-related literature, and local clinical needs. The study combined theoretical training, simulation practice, bedside mentoring, protocol standardization, and continuous feedback, which allowed nurses to apply evidence directly in clinical practice. Another strength is that the evaluation included provider-level, patient-level, and system-level outcomes, giving a broader understanding of the intervention effect. The study was also conducted in a real clinical obstetric ward, which increases the practical relevance of the findings.

### 4.2 Limitations

This study has important methodological limitations. First, the single-group pre–post design without a concurrent control group cannot exclude secular trends, changes in staffing, increased awareness of breastfeeding practices, or other co-interventions as explanations for the observed changes. Therefore, causal conclusions regarding intervention effectiveness cannot be made.

Second, the nurse sample was very small (*n* = 11), no formal sample-size calculation was performed, and the study was conducted in one tertiary hospital. These factors limit statistical power, estimate precision, and generalizability to other institutions and settings. Third, several outcomes were assessed using locally adapted observation checklists. Although these tools underwent expert review, pilot testing, and observer standardization, formal psychometric validation and inter-rater reliability testing were not performed. Measurement error and observer bias therefore remain possible. In addition, participants knew that breastfeeding practice was being observed, which may have contributed to a Hawthorne effect. The follow-up period was limited to hospitalization; therefore, sustained nurse competence and exclusive breastfeeding after discharge could not be evaluated. Future research should use adequately powered multicentre controlled designs, validated assessment instruments, independent blinded assessors where feasible, and longer postpartum follow-up.

### 4.3 Clinical and practical implications

Although the findings are preliminary, they suggest several potential clinical and policy implications:

- Competency-based breastfeeding training may be considered for adaptation and evaluation in health-care settings
- Knowledge translation strategies should be used to embed clinical practice guidelines into practice.
- Establishing standardized processes can enhance the quality of care.
- Postpartum support should include follow-up and multiple counselling sessions.
- Multidisciplinary teamwork between nurses, doctors and lactation consultants can improve care for complex breastfeeding issues.

## 5 Conclusion

This study suggests that implementation of a structured, competency-based, evidence-informed breastfeeding counselling program was associated with higher observed breastfeeding counselling practice indicators, higher maternal breastfeeding ability levels, and increased exclusive breastfeeding during hospitalization. However, because this was a single-center uncontrolled pre–post study with a small nurse sample, independent maternal assessment samples, and short follow-up, the findings should be interpreted cautiously. Larger controlled multicenter studies using validated tools and paired nurse-level data are needed to confirm effectiveness and sustainability.

## Data Availability

The original contributions presented in the study are included in the article/Supplementary Material, further inquiries can be directed to the corresponding author.
